# Derangements, Primary and Reflex, of the Organs of Digestion

**Published:** 1841-01

**Authors:** 


Art. VI.—*Derangements, primary and reflex, of the Organs of Digestion.* By Robert Dick, m.d.—*Edinburgh*, 1840. 8vo, pp. 382.

This is one of a class of books becoming common, and, as such, characteristic of an age when all subjects are discussed in print, and redd by those who have the requisite means, inclination, and leisure. It is a medical work, written under the impression that the public would peruse it, and, for their benefit, common terms are occasionally explained, prescriptions are translated, and (to use the author's own words) "episodical and divergent discussions" are indulged in. Indeed, Dr. Dick suspects that the book may get into the hands of the boys and girls of this reading, inquisitive generation, "potest accidere ut hic liber veneat in manus juvenum utriusque sexus," and, on this account, he veils in a garb of medical Latin some very useful observations. In former times poets alone had such youthful readers, but now a writer on indigestion may say

"Virginibus, puerisque canto."

Owing to this double object, the author becomes unnecessarily explanatory for his medical reader, and, consequently, he is often tedious. Few have time enough to pick out what is new from so much that is well known—to wade through an octavo volume instead of mastering a pamphlet. Besides this diffuseness of matter, Dr. Dick's style is destitute of conciseness; he frequently repeats the same idea (and that a very common-place one) in different words, and he seems seeking too often for the longest word and the most sounding sentence. Digestion is turned into "stomachic processes," to empty itself is "to disembogue," grief becomes "moral chagrin," &c.

But, in adducing these minor defects of composition, we do not mean to condemn the book: it is not a common clap-trap compilation of other men's opinions. Dr. Dick has evidently treated dyspepsia, and has suffered from it himself: he has thought much and read more about the disease; his powers of observation are considerable, and his practical remarks are, for the most part, judicious and moderate. His descriptions of the appearance and manner of patients suffering from dyspepsia, and of their mental condition, have an air of truth and freshness, such as those alone can produce who observe nature minutely and thoughtfully, and have, besides, adequate descriptive powers. We could have wished that Dr. Dick had given himself time enough to have written a shorter book—a treatise for his medical brethren only, giving them credit for knowing the meaning attached to the words life, death, vital principle, nervous influence, and such elementary matters; whilst, in the shortest compass, he had endeavoured to embody his own experience and reflection. It has been said by one of the writers in the Memoirs of the French Academy of Surgery, that "the increase of knowledge is not like that of other things; for as our opportunities increase, there is often a great diminution in its bulk:" a golden precept for all medical writers to meditate upon deeply.

